# Microfluidic production, stability and loading of synthetic giant unilamellar vesicles

**DOI:** 10.1038/s41598-024-64613-4

**Published:** 2024-06-18

**Authors:** Mart Ernits, Olavi Reinsalu, Naresh Yandrapalli, Sergei Kopanchuk, Ehsan Moradpur-Tari, Immanuel Sanka, Ott Scheler, Ago Rinken, Reet Kurg, Andreas Kyritsakis, Veikko Linko, Veronika Zadin

**Affiliations:** 1 Institute of Technology, University of Tartu, Nooruse 1, 50411 Tartu, Estonia; 2 Department of Colloid Chemistry, Max Planck Institute of Colloids and Interfaces, Am Mühlenberg 1, 14476 Potsdam, Germany; 3 Institute of Chemistry, University of Tartu, Ravila 14a, 50411 Tartu, Estonia; 4 Department of Chemistry and Biotechnology, Tallinn University of Technology (TalTech), Akadeemia Tee 15, 12618 Tallinn, Estonia; 5 Biohybrid Materials, Department of Bioproducts and Biosystems, Aalto University School of Chemical Engineering, Kemistintie 1, 02150 Espoo, Finland

**Keywords:** Phospholipids, Microfluidics, Membranes

## Abstract

In advanced drug delivery, versatile liposomal formulations are commonly employed for safer and more accurate therapies. Here we report a method that allows a straightforward production of synthetic monodisperse (~ 100 μm) giant unilamellar vesicles (GUVs) using a microfluidic system. The stability analysis based on the microscopy imaging showed that at ambient conditions the produced GUVs had a half-life of 61 ± 2 h. However, it was observed that ~ 90% of the calcein dye that was loaded into GUVs was transported into a surrounding medium in 24 h, thus indicating that the GUVs may release these small dye molecules without distinguishable membrane disruption. We further demonstrated the feasibility of our method by loading GUVs with larger and very different cargo objects; small soluble fluorescent proteins and larger magnetic microparticles in a suspension. Compared to previously reported microfluidics-based production techniques, the obtained results indicate that our simplified method could be equally harnessed in creating GUVs with less cost, effort and time, which could further benefit studying closed membrane systems.

## Introduction

In drug development, one of the key goals is to maximize the delivery precision of the drug as a significant amount of various side effects are caused by the drugs inadvertently affecting healthy tissues. Traditionally, drugs are directly administered to the patient and distributed throughout the organism, and thus, only a small proportion of the active molecules may reach their intended target. Therefore, it has been proposed that targeted drug delivery and release would significantly reduce the side effects by allowing the chemical to interact only when it is in the correct tissue^1^.

There exist several approaches that aim at developing more specific drug delivery systems for e.g. chemotherapy, including nanobiomedical technologies and materials such as viral nanoparticles, quantum dots, polymer nanomaterials and liposomes^2–4^. Among these, liposomes possess excellent properties for safe and versatile drug delivery owing to their high biocompatibility, biodegradability, low immunogenicity, and ability to cross the brain–blood barrier while carrying hydrophilic or hydrophobic drugs^5,6^. Liposomes are spontaneously forming spherical structures consisting of a lipid bilayer membrane and a hydrophilic lumen. They can be regarded as a type of vesicles that have been formed without the implementation of cellular pathways, usually with a diameter of up to 1 µm.

Liposomes are commonly produced using methods such as thin film hydration, reverse phase evaporation, and detergent removal^7,8^. However, these widely used methods may have several disadvantages including low reproducibility, poor homogeneity, relatively complex and tedious fabrication processes, the use of toxic compounds and harsh processing conditions, as well as poor drug encapsulation efficiency^9,10^. Compared to the conventional techniques, microfluidic approaches for producing liposomes exhibit some significant advantages, such as control over the vesicle size, improved cargo encapsulation efficiency, and higher reproducibility^11,12^. For example, microfluidic chips using hydrodynamic focusing allow for the production of liposomes with fine-tuned sizes, and chips using a double-emulsion-droplet-based approach provide excellent encapsulation efficiencies into larger vesicles^13–15^.

One of the challenges of liposomes is to deliver the payload specifically into targeted cells without premature drug release or lysosomal degradation^16^. By incorporating specific targeting compounds, such as antibodies or aptamers, into their membranes, their delivery accuracy can be significantly improved^16–18^. New methods, such as fusogenic liposomes with the ability to merge directly with the cell cytoplasm as well as other functionalized and triggerable lipid vesicles are coming increasingly into view^19–24^. Despite the numerous reports of promising liposome approaches, there is no complete understanding of the exact process of the bilayer manipulation of liposomes. One of the key difficulties of membrane studies is the nanoscopic size range of the liposomes, considering that liposomes with diameters of < 200 nm are optimally sized for many efficient in vivo drug delivery applications^25^. Due to their minuscule size, specialized high-technological equipment would be necessary to analyze the liposomes. To understand the responses of membrane bilayers to specific stimuli, it is often easier to study model systems that are observable through more common methods. Giant unilamellar vesicles (GUVs) are excellent models for investigating the properties of closed phospholipid membranes as they are large enough (> 10 μm in diameter) to be visualized and manipulated under a conventional optical microscope^26^.

Efforts to improve the techniques for GUV production that allow them to be studied as a model for vesicular drug delivery systems would be beneficial for the field. Here, we report a potentially scalable microfluidics-based method to produce synthetic monodisperse GUVs using only an adjustable negative pressure pump to drive the process. Furthermore, we characterize the stability of these GUVs by performing calcein leakage assays, and we also demonstrate the feasibility of the method in straightforward cargo loading by successfully encapsulating dye molecules, proteins, and microparticles into the GUVs. The obtained results add to the development of microfluidic GUV preparation methods that could be further harnessed in closed phospholipid membrane research and various liposome-based drug delivery platforms.

## Methods

### Materials for GUVs

The aqueous solutions used to produce GUVs were either MilliQ water- or phosphate buffered saline (PBS)-based as noted. All the aqueous solutions were prepared to contain 300 mM saccharides (sucrose and glucose) from Merck (Germany) and 0.5% w/v of Synperonic F108 (Croda International Plc, UK) to increase GUV stability unless noted otherwise. 1-octanol (Merck) was used as the lipid solvent in the oil phase solution to dissolve 5 mg/mL 1-palmitoyl-2-oleoyl-glycero-3-phosphocholine (POPC) (Avanti polar lipids, USA) by mixing them directly. The oil phase solution was stored at 4 °C and heated up to 60 °C for 30 min before starting the GUV production process.

### Microfluidic chip design and fabrication

**Figure 1:**
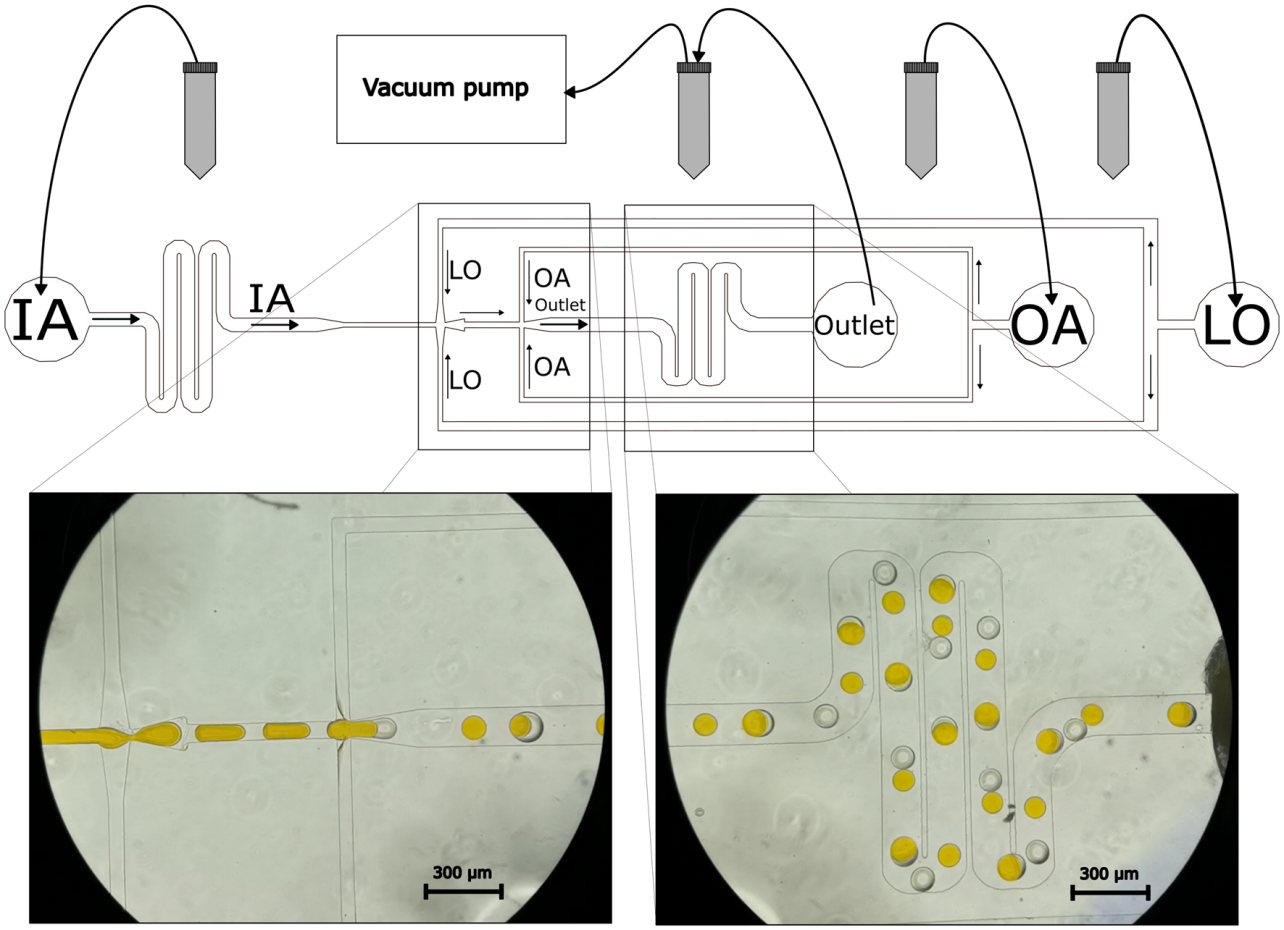
Setup of GUV production using the microfluidic chip. The inner aqueous (IA), outer aqueous (OA), and the lipid-oil (LO) solutions are pulled through the chip by a vacuum pump that is attached to the outlet tube. The vertical positions of the input reservoirs can be varied to tune the pressure ratios of the inputs. The microscopy images from the indicated areas of the chip demonstrate the formation of alternating water and oil droplets in the first junction (IA is yellow due to calcein dye) and the formation of the GUVs at the second junction.

The microfluidic production of GUVs was performed using polydimethylsiloxane (PDMS)-based chips. The chip design comprises three input channels and one outlet channel (Fig. 1). The inner aqueous (IA) solution and the phospholipid-containing oil phase (LO) solution meet in the first junction, thus forming alternating oil and water droplets into the flow channel leading into the second junction. This is where the outer aqueous (OA) solution is added to form GUVs. The chip is driven by a single adjustable vacuum pump attached to a reservoir connected to the outlet. The input solutions of the IA, OA, and LO phases are contained in reservoirs that can be raised and lowered to tune their relative pressures.

The fabrication and preparation of the chips have been thoroughly described before^15^. Briefly, 4″ silicon wafer (Siegert Wafers, Germany) molds are prepared using UV-soft lithography. Initially, SU8 (2000 series, Kayaku Advanced Materials, Inc., USA) of 80 µm thickness was spin-coated (model no. WS-650MZ-23NPPB, Laurell Tech. Corp, USA) on a plasma-cleaned silicon wafer. This was followed by a pre-baking step (65 °C for 3 min and 95 °C for 9 min) before exposing to the UV-light for 8 s (through the mask design specified in the literature^15^. After a post-baking step (65 °C for 2 min and 95 °C for 7 min), the silicon wafer was washed in a developer solution, extracting the unpolymerized SU8. The master mold was then hard-baked at 200 °C for 30 min before treating it with a silanization step (50 µL of 1H,1H,2H,2H-perfluoro-decyl trichlorosilane) overnight.

A 10:1 mixture of PDMS:curing agent (SYLGARD^®^ 184 silicone elastomer kit from Dow Silicones Corporation, USA) was poured over the master mold and cured at 90 °C for 3 h. Thus, cured PDMS was stripped from the mold, diced, and punched with a 1 mm biopsy puncher at various inlets shown in Fig. 1. These PDMS chips were bonded onto plasma-cleaned (600 mbar for 2 min) (Plasma Cleaner PDC-002-CE, Harrick Plasma, USA) glass coverslips and heated at 60 °C for 2 h before performing surface passivation for double emulsion production. The surface passivation of the hydrophobic PDMS chips are crucial for double emulsion production. A step-by-step process is presented elsewhere^27^. During this process, the second T-junction towards the outlet (Fig. 1) is flushed with oxidizing solution (HCl:H_2_O_2_—1:2) and then the channels were coated with a positively (10 wt%, poly(diallyldimethylammonium chloride, Sigma Aldrich, Germany) and negatively charged (2 wt%, poly(sodium 4-Styrenesulfonate, Sigma Aldrich) polymers, sequentially. This polyelectrolyte layer-coated channel is thus rendered hydrophilic for double emulsion production.

### GUV production and loading process

The microfluidic PDMS chip connected to inlet and outlet reservoirs was placed under an optical microscope (Eclipse TS-100, Nikon Instruments Inc., USA) and the negative pressure in the outlet channel was generated using a vacuum pump (VG350, Dolomite Microfluidics, UK). When the solution from the OA input to the Outlet was seen to flow steadily, the LO and IA inputs were added. The vacuum pressure in the chip was first varied to get rid of air bubbles and then set to ~ 20 mbar to start the GUV production process. The vertical positions of LO and IA reservoirs were adjusted (raised or lowered) to create a proper alternating and balanced flow of water and oil droplets from the first junction. Once the desired flow was achieved, the GUVs started to form at the second junction. Then the vacuum pressure was increased, and the LO container was raised gradually to achieve faster GUV production rates. The flow of GUVs through the outlet tubing was monitored to make sure that all the waste material (a mixture of the inlet solutions without any GUVs from the initiation of the process) had flowed through it. Once this stage was reached, the outlet was switched to a clean centrifuge tube without interrupting the vacuum. Using a vacuum pressure of 32 mbar, ~ 1.5 mL of the GUV suspension was produced within ~ 30 min. The production of the GUVs was constantly monitored, and the images and slow-motion movies (Supplementary Movies SV1 and SV2) of the chip during the procedure were captured at 1920 frames per second with a high-speed camera (Huawei Technologies Co., Ltd., China).

To demonstrate magnetic microparticle loading into GUVs, COMPEL™ Fluorescent Magnetic COOH, Microspheres, Glacial Blue (Bangs Laboratories, USA) with a diameter of 3.2 μm (blue magnets) were used. The particles were diluted 1:1 with the inner solution, resulting in an IA of 150 mM saccharides and 0.25% Synperonic F108 dissolved in MilliQ water with 0.5 mg/mL of the particles. The OA solution was also diluted 1:1 using MilliQ water to match the IA osmolarity. The magnetic nanoparticle loaded GUVs were imaged using a confocal microscope (LSM710, Zeiss, Germany). To produce enhanced green fluorescent protein (eGFP) loaded GUVs, MilliQ water-based IA solution was supplemented with 50 ng/μL of eGFP. The protocol for eGFP production and purification from the bacterial expression system has been described earlier^28^. The produced GUVs were imaged with a fluorescence microscope (EVOS M5000, Thermo Fisher Scientific Inc., USA).

### Microscopic characterization of the GUV stability

For the visual stability assessment of the GUVs, the vesicles were produced using a PBS-based IA solution containing 5 mM of calcein (Merck). A sealed hybridization chamber of 30 μL on a microscope slide was filled with the freshly prepared GUV suspension. Bright-field and epifluorescence scans of whole slides were performed at different time points using an inverted microscope built around a Till iMIC body (Till Photonics/FEI, Munich, Germany). Images were acquired using a PlanFLN 10× (NA 0.3) objective (Olympus Corp., Tokyo, Japan). In the emission path, an additional 2× magnification was provided by a TuCam dual camera adapter (Andor Technology, Belfast, UK). Light detection was performed using an iXon Ultra 897 EMCCD camera (Andor Technology, Belfast, UK). For epifluorescence imaging, the probes were excited with a 488-nm PhoxX laser diode (Omicron-Laserage, Rodgau, Germany) and the emitted light was spectrally separated with a ZT488/561rpc polychromatic dichroic mirror (2.0 mm substrate, Chroma Technology, Bellows Falls, USA) and a BrightLine 524/628-nm dual-band bandpass filter (Semrock Inc., Rochester, USA). The entire workflow was organized using a tiling protocol with autofocusing in Live Acquisition 2.7 software (FEI, Munich, Germany). The obtained images were stitched together using the Microscopy Image Stitching Tool (MIST) plugin for the open-source software named ImageJ. The plugin was configured to assume a 20% overlap between the images with an uncertainty of 1%.

The GUVs were counted from the stitched fluorescent channel images using an AI-assisted approach. The machine learning model was trained by Yolov5 code with a dataset containing 60 images with 640 × 640 pixel dimensions. The batch size was 32 images, whereas 40 images were used for training and 20 for validation. The detection training was done by a deep neural network containing 379 layers with a learning rate of 0.01 and one class. In the neural network model, the Leaky ReLU activation function was used in the middle/hidden layers, and the sigmoid activation function was used in the final detection layer. Stochastic gradient descent was also employed as an optimization function. After training, a detection precision of about 95% was achieved. An example of the AI-assisted GUV detection is presented in Supplementary Fig. S1.

### Calcein release assessment for the GUV stability

To perform quantitative measurements of calcein release from the produced GUVs, the relative amount of calcein in the GUV-containing solution was assessed using a fluorometric approach adapted from Lajunen et al.^29^. GUVs were prepared with a self-quenching concentration of calcein (60 mM) in the PBS-based IA solution. The sample of calcein-loaded GUVs was diluted 1:50 with the corresponding OA solution that had been treated by mixing it with LO and removing the excess oil phase. This was to maintain the properties of the GUV medium because it was found that without the LO treatment, the liposomes would start to rapidly break at dilutions of 1:7 using untreated OA. Next, the GUV suspension was divided into 12 aliquots of 100 µL on a 96-well microtiter plate and sealed with a plastic cover. The samples were kept at room temperature throughout the experiment. The fluorescence intensity of the samples was measured using a Synergy Mx (Bio-Tek Instruments, Inc., USA) microplate reader. The fluorescence intensity was recorded every 60 min using a filter set with excitation and emission bandwidths of 485/9.0 nm and 526/20.0 nm, respectively. As a control, the calcein/IA solution was diluted in the same way as the GUV containing samples. The mean fluorescence intensity values of the control samples were used to normalize the fluorescence intensity values of the GUV samples at each time point to exclude other factors affecting the calcein fluorescence intensity.

After measuring the fluorescence intensity of the released calcein for at least 60 h, the aliquots were treated with 0.5% Triton-X (Merck) to degrade any remaining GUVs. The fluorescence intensity value obtained after the treatment was considered to correspond to the complete release situation (100% of calcein released).

Based on this data, the calcein release percentage at a given time was calculated using the following formula:

1 $$R = \frac{{F - F_{0} }}{{F_{100} - F_{0} }} \times 100\% ,$$

where* R* is the calcein release percentage, *F* the fluorescence intensity at the given time point, *F*_0_ the fluorescence intensity at the time point zero, and *F*_100_ the fluorescence intensity after all the GUV membranes were degraded using Triton X-100.

## Results

### GUV production using a microfluidic chip

**Figure 2:**
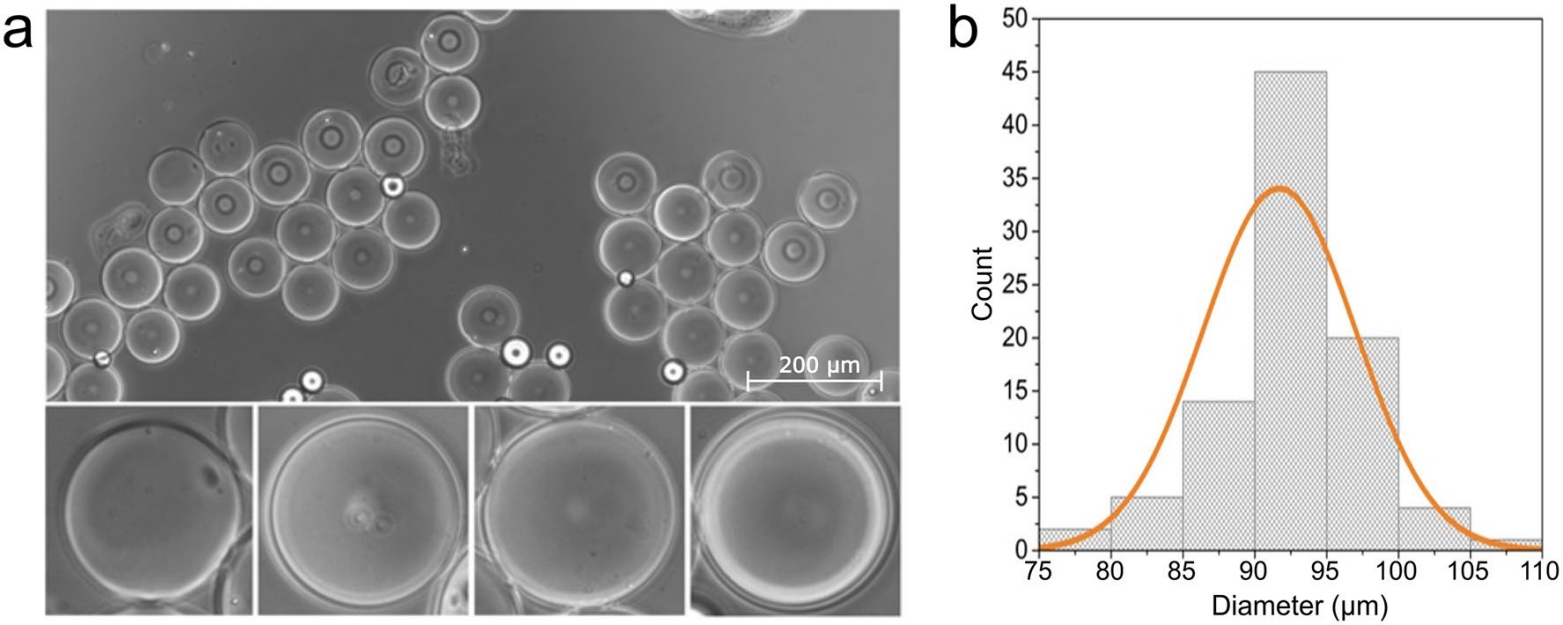
Produced GUVs. (**a**) A bright-field microscopy image of the produced GUVs on a microscopy slide. Zoomed-in insets are 100 × 100 μm^2^ in size. (**b**) Distribution of the diameters of the GUVs (*n* = 91) of this production batch, an average diameter of a GUV is 92 ± 6 μm (s.d.). Each bar on the graph represents the relative amount of GUVs with diameters in a range of 5 μm. The Gaussian fitting of the data is shown in orange.

We used a microfluidic chip utilizing the water-in-oil-in-water emulsion (W/O/W) approach to produce the GUVs. The GUVs consisted of a simple POPC monolipid bilayer that was formed by emulsifying lipid-octanol solution between the IA and OA solutions. Previously, due to three different inlet channels and one outlet channel, three independent pressure adjustable pumps were required^15^. Here, the approach was made less complex, as the GUVs were produced by employing only a single adjustable negative pressure pump at the outlet channel (Fig. 1). Examples of GUV formation in the microfluidic chip are shown in Supplementary Movies SV1–SV2 (for both empty GUVs and loaded GUVs). A representative bright-field microscopy image of the produced GUVs on a microscopy slide is presented in Fig. 2a. The crude suspension included some GUVs with noticeable amounts of solvent in the bilayer. There are also small droplets of 1-octanol as a side product formed beside the GUVs. 1-octanol may have separated from the GUV membranes or have been released due to the degradation of some of the GUVs. Nevertheless, the produced GUVs are rather monodisperse in size, and in this particular production batch (from Fig. 2a) the average diameter of GUVs was 92 ± 6 μm (s.d., *n* = 91) as shown in Fig. 2b.

### The stability of the GUVs

**Figure 3:**
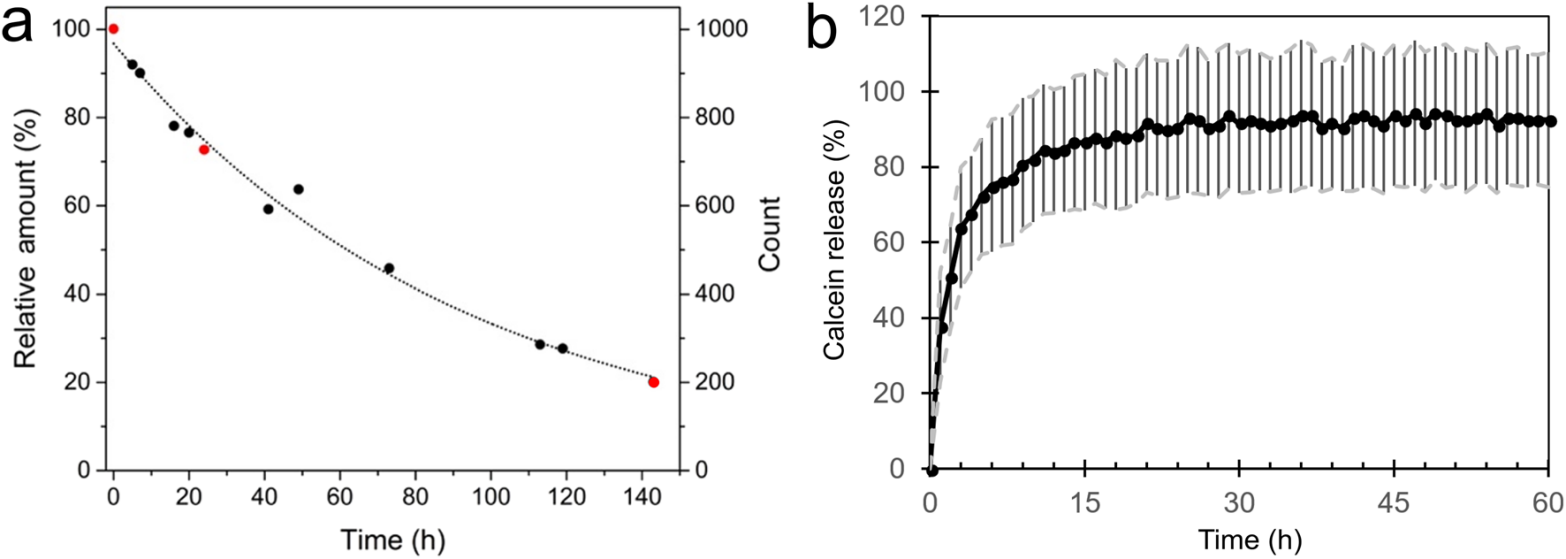
Liposome stability. (**a**) Relative amount of GUVs in a chamber over time (100% GUVs at *t* = 0). The number of GUVs vs. time follows an exponential decay curve with a half-life of the GUVs being ~ 61 ± 2 h (SEM). The three time points marked in red correspond to the images shown in Supplementary Fig. S2. (**b**) Calcein release over time was measured via fluorescence assay. 90% of the loaded calcein is released during the first 24 h of the experiment.

The stability of the GUVs was assessed using two different approaches: (1) visually through microscopic imaging and (2) fluorometrically by analyzing calcein release from the GUVs. Firstly, the visual observation of the stability entailed storing the GUVs at room temperature in a sealed chamber and counting the GUVs at different time points. The GUVs were loaded with calcein at the concentration that gives the highest fluorescence intensity (5 mM), which thus enabled straightforward particle recognition for AI-assisted GUV counting from the images. Most of the GUVs formed a single large group which gradually decreased in size over time (Supplementary Fig. S2). During a period of ~ 6 days, the number of GUVs decreased by ~ 80% (Fig. 3a). The number of GUVs was found to obey an exponential decay with a corresponding half-life of 61 ± 2 h (SEM). It is noteworthy that the samples dried to some extent over the course of the experiment. This could be observed as the air bubbles grew inside the chamber and the chamber appeared to shrink in depth under the atmospheric pressure as the sample volume decreased (see Supplementary Fig. S2). The experiment was terminated well before the available space for the GUVs started running out, however the drying-induced change in the osmotic pressure may have affected the stability of the GUVs as well.

For the fluorometric approach, the GUVs were loaded with a self-quenching concentration of calcein (60 mM) and the fluorescence intensity of the surrounding medium was monitored. The GUVs were stored in sealed wells of a microtiter plate at room temperature. Surprisingly, there was a striking difference between the stability results obtained using these two approaches. The fluorometric measurements suggested that the calcein was leaking rapidly from the GUVs, as ~ 90% of the calcein was already released during the first 24 h of the experiment (Fig. 3b). It could be speculated that the pocket of air sealed together with the sample into the microtiter plate wells could have affected the liposome stability as the oily liposomes tend to rise close to the surface of the suspension. At the surface, drying or direct contact with air may have contributed to the disruption of the GUVs. Although the GUV sample for visual assessment was loaded into the chamber without large air pockets, drying was also observed as air bubbles formed and grew. The presence of Synperonic F108 as a potential edge-activator could make the membrane of the GUVs more flexible, and in turn, it could also make the vesicle prone to a cargo leakage^30^. Therefore, the calcein might have also been released from intact GUVs and this would have increased the overall fluorescence intensity of the sample faster than what the liposome counting experiment would suggest.

### Loading GUVs with magnetic microparticles and fluorescent proteins

**Figure 4:**
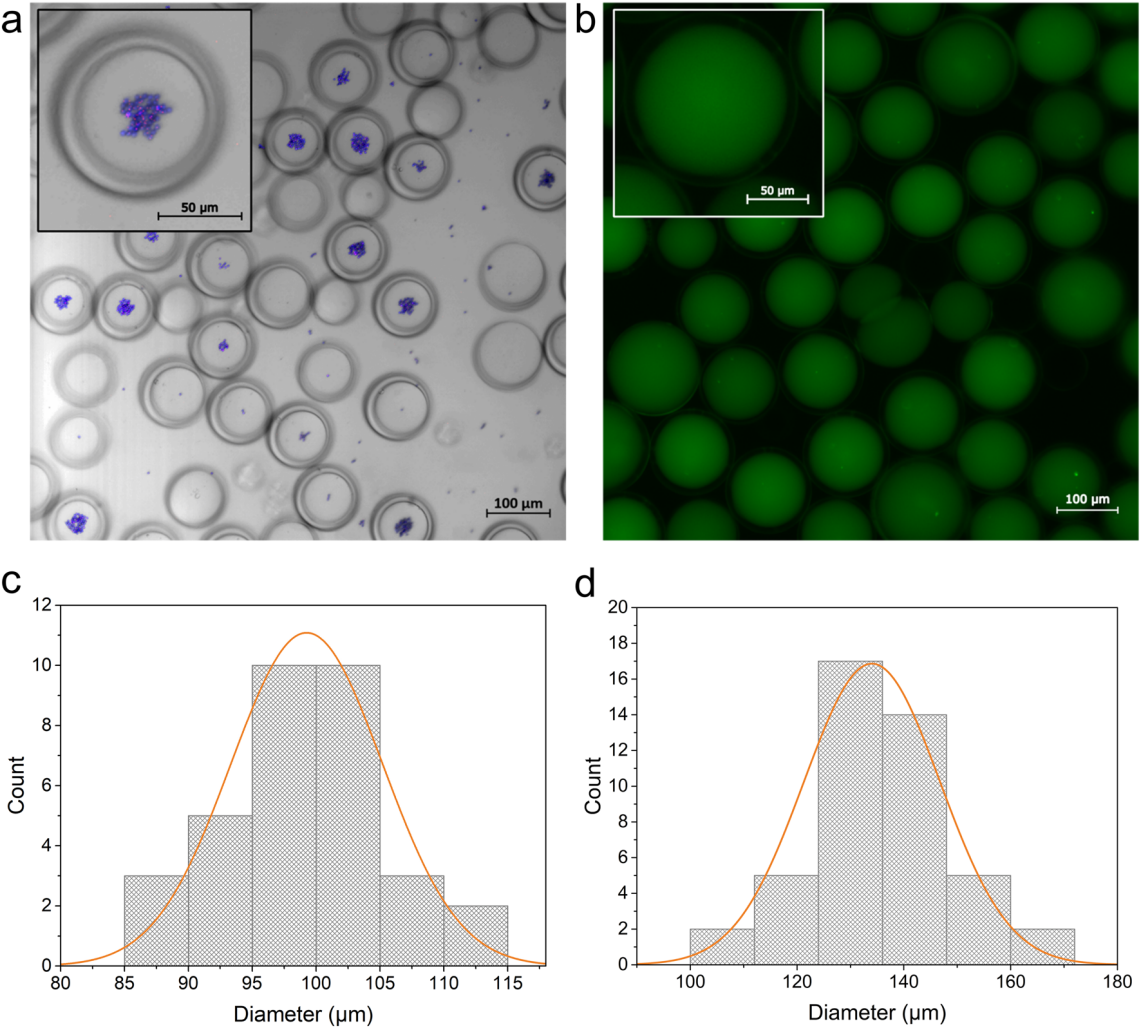
GUVs loaded with various payloads. (**a**) Confocal microscopy image of GUVs loaded with Glacial Blue-coated fluorescent magnetic microparticles. (**b**) Fluorescence microscopy image of GUVs containing eGFP. (**c**) Distribution of the diameters of liposomes loaded with magnetic microparticles (*n* = 33) with an average diameter of 99 ± 6 μm. (**d**) Distribution of the diameters of liposomes loaded with eGFP (*n* = 45), the average diameter is 134 ± 13 μm. Each bar on the graphs represents the amount of GUVs with diameters in a range of 5 μm in (**c**) and 12 μm in (**d**). The Gaussian fittings of the data are shown in orange.

By changing the composition of the IA solution, the contents of the liposomal lumen can easily be altered. This allows producing GUVs with strictly defined interior properties and cargo. We showed that the GUVs were loaded with a fluorescent dye, calcein, but to study the GUV loading capability of the chip further, two different payloads—magnetic microparticles in a suspension with a nominal diameter of ~ 3.2 µm and soluble eGFPs were incorporated into the GUVs during the vesicle production process (Fig. 4). The relatively large and heavy magnetic particles tended to sediment rapidly and were loaded into the GUVs unevenly. Similar behavior has been previously observed with polystyrene microspheres^15^. In Fig. 4a, the magnetic particles inside GUVs are in focus similarly to the particles dispersed onto the microscopy slide surface. This suggests that the particles are stacked on the bottom of the GUVs. In turn, due to the good solubility of the eGFP, the proteins got uniformly distributed inside the GUVs, as shown in Fig. 4b. From this image, it is also possible to discern the thick oil layers and the eGFP-containing lumens of the GUVs. In Fig. 4c,d, the size distributions of the liposomes from Fig. 4a,b are shown, respectively.

## Discussion

In general, microfluidics is considered one of the most promising high-throughput techniques for the versatile production of uniform drug-encapsulating liposomes. The method equally allows for the use of model systems, such as GUVs, with similar characteristics. However, the initial costs related to the fabrication equipment and chip manufacturing may make the approach less attractive. These microfluidic chip designs are often based on multi-channel architectures, which require independent pumping units to provide appropriate pressures in each channel of the chip. Good quality microfluidic pressure control equipment enabling precise individual multi-channel pressure regulation may be very expensive. Therefore, steps to decrease the need for such devices would render the approach more accessible. The method shown here demonstrates that GUVs can be produced in a multi-channel chip by generating negative pressure at the outlet channel only. Moreover, the produced GUVs were rather monodisperse, indicating the feasibility of the simplified setup.

One of the drawbacks of the W/O/W approach in microfluidic GUV production is the oiliness of the crude output^13,31^. Moreover, employing only a single negative pressure pump as the driving unit provides less stable control over the individual inlet pressures resulting in the GUVs often having noticeably thicker membranes due to entrapped solvent between the outer and inner phospholipid leaflets. This is well illustrated by the fact that the average GUV diameters varied between different production batches, as can be seen from Figs. 2b, 4c,d. Nevertheless, the monodispersity is high in all individual batches. The 1-octanol should be able to eventually escape from the vesicle membrane^15^, but this would in any case leave the solvent present in the GUV suspension. By employing a minimal amount of 1-octanol, the escaped 1-octanol may dissolve in the surrounding water of the suspension, but if it is used at higher concentrations, it may accumulate into droplets or large blobs. Therefore, additional steps may be required to purify the GUVs of the solvent when using the presented method.

Although GUVs are generally considered more stable than smaller unilamellar vesicles due to their lower curvature^32^, it is important to be aware of the limitations the GUVs may have, including their shelf-life. Interestingly, we observed that calcein was released from the GUVs at a much faster rate than the visually confirmed vesicular integrity would suggest. This might indicate that the produced GUVs are leaky and able to release small dyes without distinguishable membrane disruption. Nevertheless, in ambient conditions, the GUVs remained rather stable as there were still more than 70% of the GUVs left after 24 h of incubation, and about a fifth of the traced population survived for at least six days. The conditions of the performed experiments do not allow for prolonged storage of the GUVs but rather enable experimental studies within a few days’ time frame.

Ultimately, the successful demonstration of GUV production using microfluidics paves the way for creating stimuli-responsive or fusogenic liposomes for accurate drug delivery. Easily observable GUVs allow accessible examination of the vesicles to study the effect of functionalization on the vesicular membrane and the transfer of drugs between membrane-bound systems, including cell-mimicking vesicles^33^. Here we showed that our method allows the loading of GUVs with soluble biomolecules and suspended inorganic particles of various sizes. We incorporated calcein as a fluorescent dye into GUVs, and used a reporter protein common in biological research, eGFP, as a model protein cargo. In addition, magnetic microparticles were encapsulated by GUVs to further emphasize the capability of the technique. The integration of magnetic nanoparticles could also open opportunities to produce magnetoliposomes in future studies. Magnetoliposomes have been proposed as one solution to enable external magnetic field-triggered spatiotemporal drug release, and they have also found uses as magnetic resonance imaging contrast agents^20,34–36^. GUVs loaded with magnetic particles could serve as beneficial models for studying the effects these magnetic particles may have on liposomal membrane integrity. These model systems could also increase understanding of the conditions required to improve the accuracy and effectiveness of drug release from them, and the overall stability of magnetoliposomes.

### Supplementary Information

Supplementary Information.

Supplementary Video 1.

Supplementary Video 2.

## Data Availability

All data generated or analyzed during this study are included in this published article and its Supplementary Information files. Raw data files are available from the corresponding authors upon reasonable request.
