# Supplementary material for: Microfluidic production, stability and loading of synthetic giant unilamellar vesicles
Source: Sci Rep. 2024 Jun 18;14:14071. doi: 10.1038/s41598-024-64613-4 (PMC11189546; doi:10.1038/s41598-024-64613-4)
Supplement: Supplementary file 1 — Supplementary Information. [file 41598_2024_64613_MOESM1_ESM.pdf]

Supplementary Information

Microfluidic production, stability and loading of synthetic giant unilamellar vesicles

Mart Ernits[1], Olavi Reinsalu[1], Naresh Yandrapalli[2], Sergei Kopanchuk[3], Ehsan Moradpur-Tari[1], Immanuel Sanka[4], Ott Scheler[4], Ago Rinken[3], Reet Kurg[1], Andreas Kyritsakis[1,*], Veikko Linko[1,5,*], and Veronika Zadin[1,*]

[1] Institute of Technology, University of Tartu, Nooruse 1, 50411 Tartu, Estonia

[2] Max Planck Institute of Colloids and Interfaces, Department of Colloid Chemistry, Am Mühlenberg 1, 14476 Potsdam, Germany

[3] Institute of Chemistry, University of Tartu, Ravila 14a, 50411 Tartu, Estonia

[4] Department of Chemistry and Biotechnology, Tallinn University of Technology (TalTech), Akadeemia tee 15, 12618 Tallinn, Estonia

[5] Biohybrid Materials, Department of Bioproducts and Biosystems, Aalto University School of Chemical Engineering, Kemistintie 1, 02150 Espoo, Finland

*Emails: andreas.kyritsakis@ut.ee; veikko.pentti.linko@ut.ee; veronika.zadin@ut.ee

AI-assisted GUV recognition on microscopy images

Counting of the GUVs from the stitched images was carried out using AI-assisted approach as described in the Methods section. An example of the AI recognition of GUVs is shown in **Figure S1**.

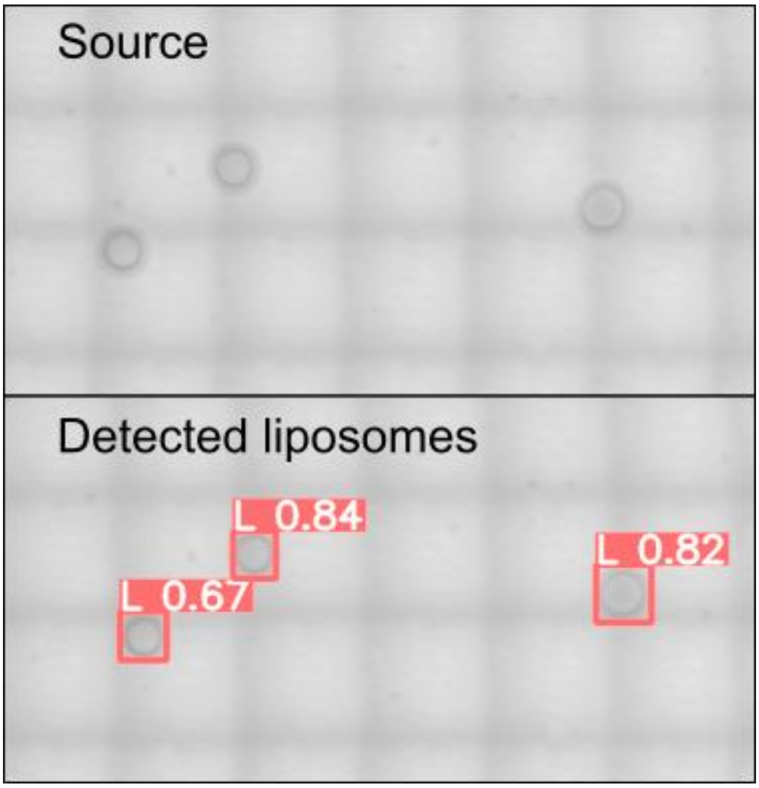

Figure S1. The detection of liposomes from microscopy images using AI. The top panel is an exemplary cutout region of the original stitched image before AI-assisted counting. The bottom panel shows the detected GUVs by the analysis in this region.

Stability assay based on microscopy imaging

Most of the GUVs loaded to the imaging chamber formed a large group that stayed together until the termination of the experiment as shown in **Figure S2** (these selected time points are marked in red in the main article **Figure 3a).** The size of the group of GUVs slowly diminished over time. Simultaneously, the air bubbles inside the chamber grew larger.

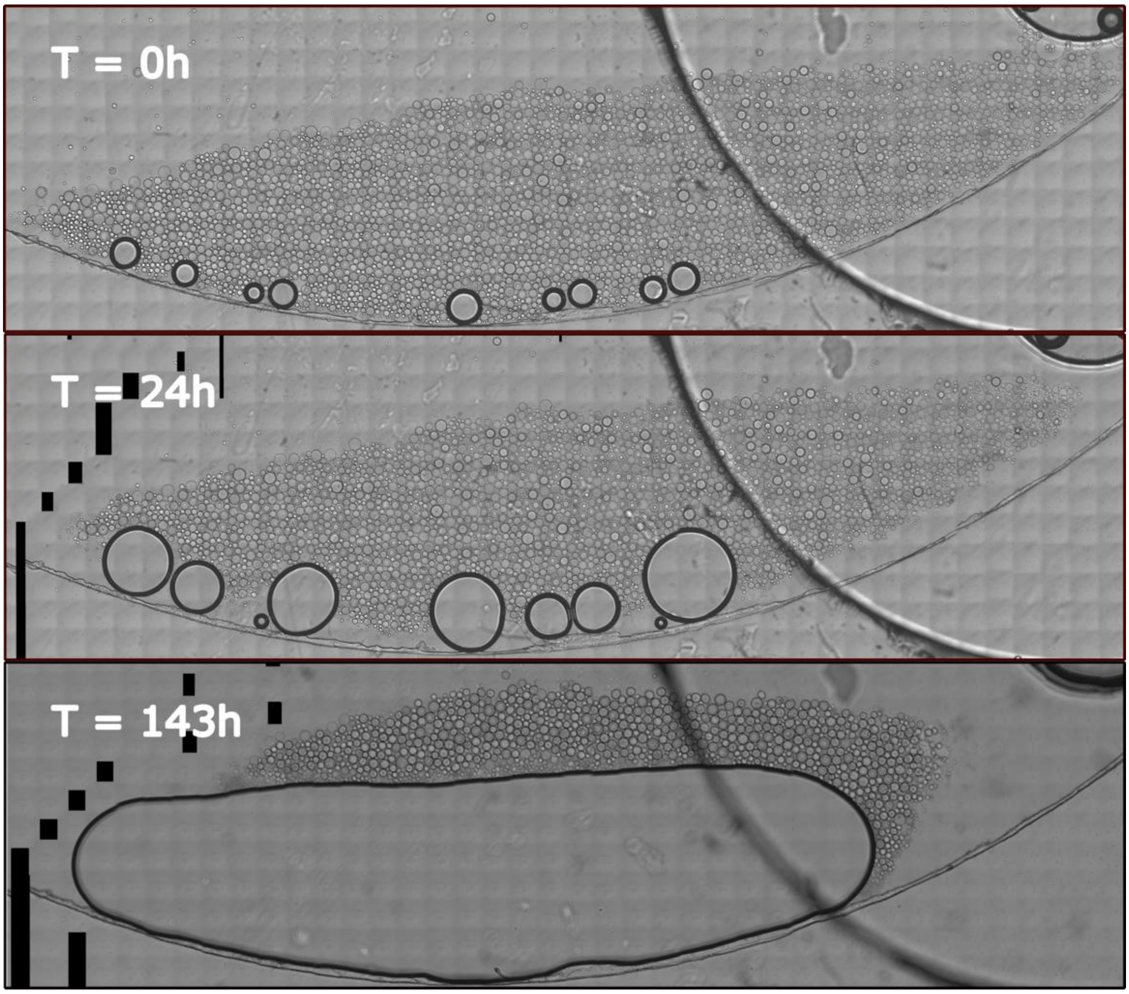

Figure S2. The population of vesicles shrinks through time. The three panels (stitched images) depict the large group of GUVs in the chamber at different time points. The black rectangles are artifacts from inaccuracies in the image stitching process.

Production and flow of GUVs in the microfluidic chip

The production of the GUVs in the microfluidic chip was captured using a high speed camera. The formation of the vesicles can be observed in the **Movies SV1** and **SV2.** The movies were recorded partially at 1920 frames per second, while the beginning and ending sections were captured at 30 frames per second.

Movies SV1 and **SV2** are provided as separate files in the article webpage.
